# Induced neural progenitor cell‐derived extracellular vesicles promote neural progenitor cell survival via extracellular signal‐regulated kinase pathway

**DOI:** 10.1111/cns.13744

**Published:** 2021-10-13

**Authors:** Yizhao Ma, Xiaonan Xu, Chunhong Li, Yi Wang, Jie Zhu, Xiaohuan Xia, Jialin C. Zheng

**Affiliations:** ^1^ Center for Translational Neurodegeneration and Regenerative Therapy Shanghai Tenth People’s Hospital affiliated to Tongji University School of Medicine Shanghai China; ^2^ Translational Research Institute of Brain and Brain‐Like Intelligence Shanghai Fourth People's Hospital affiliated to Tongji University School of Medicine Shanghai China; ^3^ Collaborative Innovation Center for Brain Science Tongji University Shanghai China

## Abstract

Proposed model for the anti‐apoptotic effects of induced neural stem/progenitor cell (iNPC)‐derived extracellular vesicles (EVs). iNPC release EVs that are abundantly loaded with growth factor‐related proteins. These growth factor‐enriched EVs enhance the phosphorylation of extracellular signal‐regulated kinase (ERK), but not AKt. The EV‐induced activation of ERK pathway then inhibit the apoptosis of NPCs in various pathological conditions including oxidative stress and starvation.

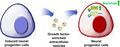

## CONFLICT OF INTERESTS

The authors declare non‐financial conflict of interest.

Extracellular vesicles (EVs) are phospholipid‐bilayer‐enclosed extracellular spherical structures that regulate a variety of cellular processes by horizontally transferring bioactive cargos.^1^ Emerging evidence has implied neural stem/progenitor cell (NPC)‐derived EVs (WT‐EVs) as promising therapeutic strategy to replace stem cell transplantation in treating neurological disorders, especially for neurodegenerative diseases like Alzheimer's disease (AD) that are in lack of effective therapeutic drugs.^2^ Unfortunately, the application of NPCs in mass production of EVs are restricted due to ethical/religious concerns, problematic logistics of acquiring fetal tissues, and potential autogenous immune response.^3^ Hence, we reprogrammed somatic cells into induced NPCs (iNPCs) to avoid aforementioned issues.^4^ Interestingly, EVs released from both fibroblast‐derived iNPCs (F‐EVs) and astrocyte‐derived ones (A‐EVs) exhibit higher potential in promoting NPC proliferation *in vitro* versus WT‐EVs.^5^ However, the roles of those EVs on neuroprotection remain unknown. We, therefore, examined the effects of NPC‐ and iNPC‐derived EVs on NPC survival *in vitro*. Data are presented as the means ±SEM. Shapiro‐Wilk tests were used to evaluate the normality of the distribution. One‐way ANOVA or Kruskal‐Wallis ANOVA were used for data that are normally distributed or not normally distributed, respectively. Significance was set at *p* < 0.05.

**FIGURE 1:**
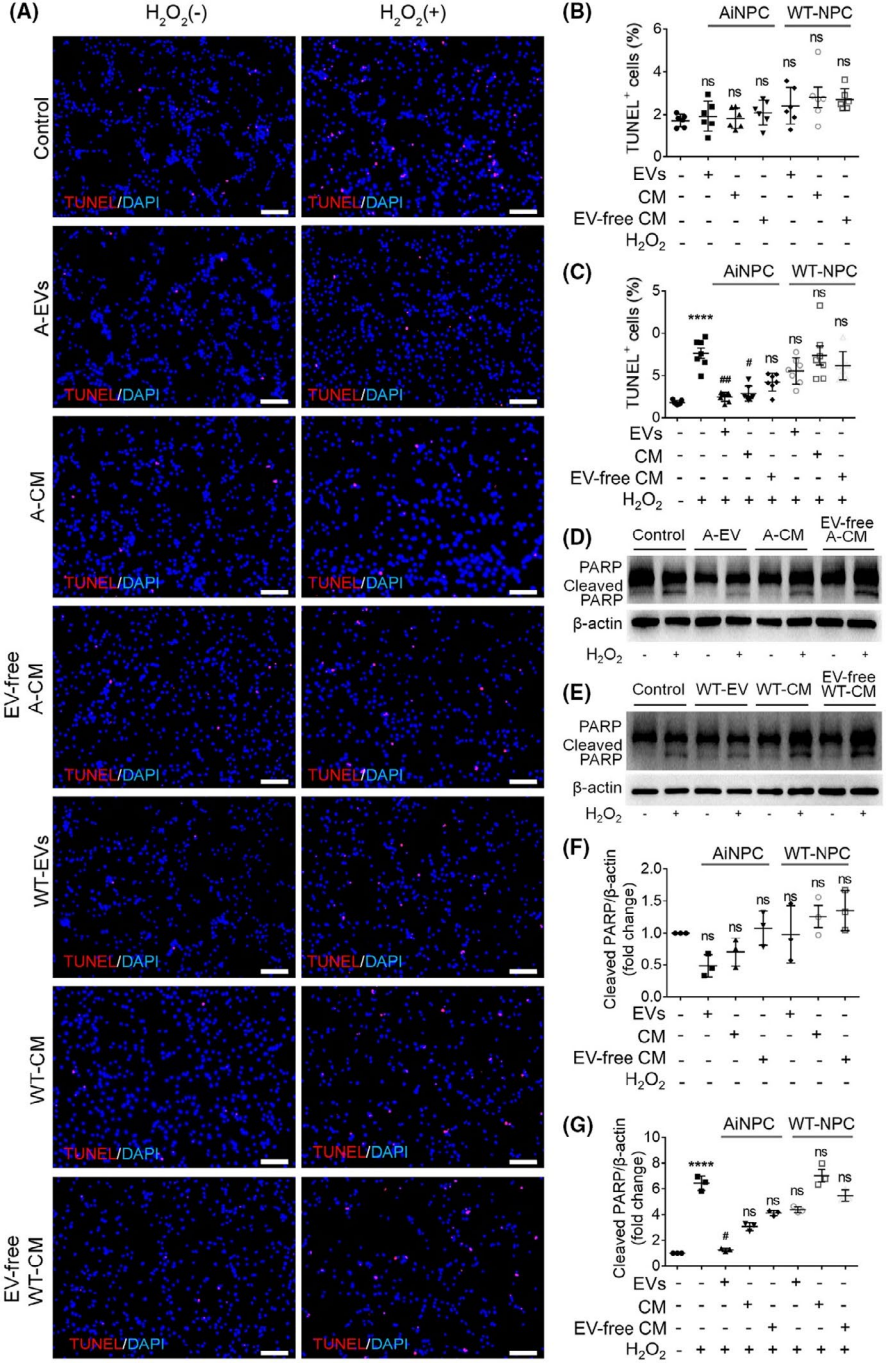
EVs are the central anti‐apoptotic secretomes of iNPCs. A, NPCs were treated with 50% CM, 50% EV‐free CM, or 15 μg/ml EVs derived from either NPCs or iNPCs for 3 hours under normal and oxidative stress conditions. Representative images of TUNEL (red) and DAPI (blue) staining were shown. B, Quantification of TUNEL^+^ cells (as a percentage of total cells) in normal culture condition. C, Quantification of TUNEL^+^ cells (as a percentage of total cells) in H_2_O_2_‐treated culture condition. D, The representative western blots showing the expression of cleaved PARP and total PARP in NPCs treated with 50% CM, 50% EV‐free CM, or 15 μg/ml EVs derived from iNPCs under normal and oxidative stress conditions. E, The representative western blots showing the expression of cleaved PARP and total PARP in NPCs treated with 50% CM, 50% EV‐free CM, or 15 μg/ml EVs derived from NPCs under normal and oxidative stress conditions. F, Quantification of expression of cleaved PARP in NPCs under normal condition. G, Quantification of expression of cleaved PARP in NPCs under oxidative stress condition. Results are presented as the mean ±SEM. Scale bar, 100 μm (A). **** denotes *p*<0.0001 in comparison with negative control group. # and ## denote *p*<0.05 and *p*<0.01, respectively, in comparison with untreated H_2_O_2_ group

To determine the roles of EVs on NPC survival, we first characterized EVs derived from NPCs and iNPCs. EVs were isolated from the conditioned medium (CM) of either NPCs or iNPCs using gradient centrifugation approach. Scanning electron microscopy indicated a great number of vesicles being released from the plasma membrane (Figure S1A). Transmission electron microscopy revealed typical cup‐shaped morphology of EVs with sizes less than 200 nm (Figure S1B). Western blotting identified EVs specific markers Flotillin‐1 and HSP70 in EV lysates, confirming the purification of EVs (Figure S1C). Afterward, 15 μg/ml EVs were added into an oxidative stress‐induced cell death model by treating NPCs with 100 μM H_2_O_2_ for 3 hours. Significantly lower proportions of TUNEL^+^ cells were observed in F‐EVs and A‐EVs treatment groups, but not in WT‐EVs treatment group, compared with untreated H_2_O_2_ group (Figure S2A,B). Western blotting also showed that the H_2_O_2_‐induced Caspase‐3 cleavage was suppressed by both F‐EVs and A‐EVs but not WT‐EVs (Figure S2C,D). Furthermore, we incubated 15 μg/ml EVs with NPCs in a nutrient deprivation‐induced cell death model by culturing NPCs in growth factors‐free medium for 12 hours. The results showed that iNPC‐derived EVs, but not WT‐EVs, significantly reduced the proportion of starvation‐elevated TUNEL^+^ cells (Figure S3A,B) and the expression levels of cleaved PARP (Figure S3C,D), confirming the anti‐apoptotic effects of iNPC‐derived EVs. Since NPCs release various secretomes with bio‐functions, we next clarified that whether CM and EV‐free CM had similar anti‐apoptotic effects as EVs. TUNEL assay indicated that EVs, CM, and EV‐free CM had no effect on apoptosis under normal conditions (Figure 1A,B). Only A‐EVs and astrocyte‐derived iNPC‐CM (A‐CM) exhibit significant effects in inhibiting apoptosis under oxidative stress condition, ascertained by the reduction of the proportions of TUNEL^+^ cells in A‐EV and A‐CM treatment groups versus untreated H_2_O_2_ group (Figure 1A,C). Similarly, western blotting results showed that EVs, CM, and EV‐free CM had no effects on the cleavage of PARP under normal conditions (Figure 1D‐F). Under H_2_O_2_ treatment condition, only A‐EVs and A‐CM significantly repressed the production of cleaved PARP, compared with untreated H_2_O_2_ group (Figure 1D,E,G). Together, our results suggested iNPC‐derived EVs as the main iNPC secretomes in promoting NPC survival.

**FIGURE 2:**
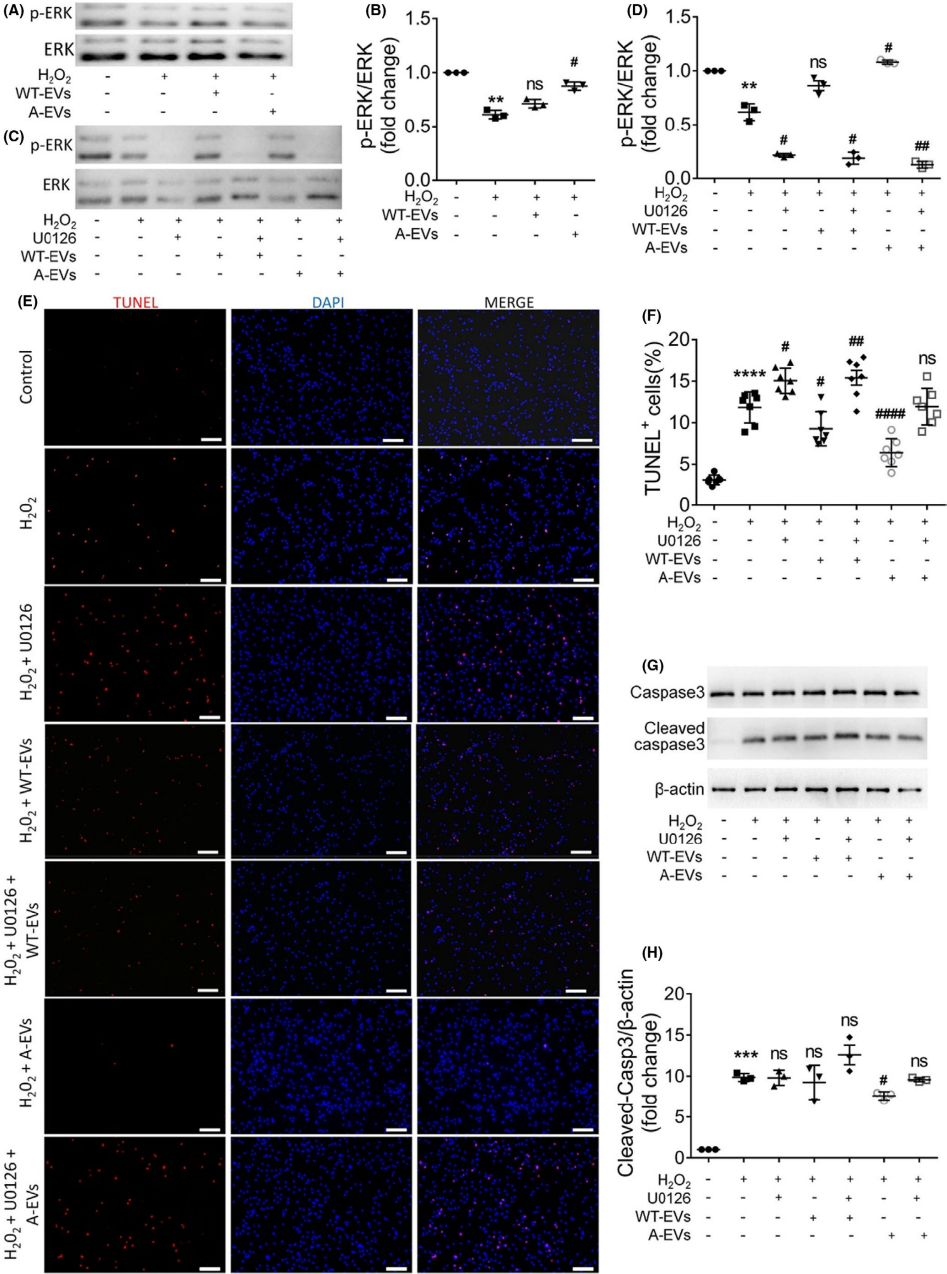
ERK pathway mediates the anti‐apoptotic effects of iNPC‐derived EVs. A, B, The representative western blots showing the expression of phosphorylated ERK (p‐ERK) and total ERK in NPCs treated with 15 μg/ml WT‐EVs or A‐EVs for 3 hours in a H_2_O_2_‐induced *in vitro* apoptosis model (A). Densitometric quantifications of the p‐ERK were demonstrated on the right panel (B). C, D, NPCs were treated with 15 μg/ml WT‐EVs/A‐EVs and 10 μM U0126 for 3 hours in a H_2_O_2_‐induced *in vitro* apoptosis model. The representative western blots showing the expression of phosphorylated ERK (p‐ERK) and total ERK in NPCs treated with 15 μg/ml WT‐EVs/A‐EVs and 10 μM U0126 for 3 hours in a H_2_O_2_‐induced in vitro apoptosis model were presented (C). Densitometric quantifications of the p‐ERK were demonstrated on the right panel (D). E, Representative images of TUNEL (red) and DAPI (blue) staining were shown. F, Quantification of TUNEL^+^ cells (as a percentage of total cells) in the culture. G, The representative western blots showing the expression of cleaved Caspase‐3 and total Caspase‐3 in WT‐EV/A‐EV‐ and U0126‐treated NPCs under oxidative stress condition. H, Quantification of expression of cleaved Caspase‐3. Scale bar, 100 μm E, Data were represented as mean ±SEM. **, ***, and **** denote *p*<0.01, *p*<0.001, and *p*<0.0001, respectively, in comparison with negative control group. #, ##, and #### denote *p*<0.05, *p*<0.01, and *p*<0.0001, respectively, in comparison with untreated H_2_O_2_ group

In previous studies, we have shown that iNPC‐derived EVs are enriched with growth factors and enhance NPC proliferation via activating extracellular signal‐regulated kinase (ERK) pathway.^5^ Here, western blotting results demonstrated that the H_2_O_2_‐induced ERK phosphorylation inhibition in NPCs could be abrogated by A‐EVs, but not WT‐EVs, suggesting the positive effects of A‐EVs in the activation of ERK pathway (Figure 2A,B). In contrast, EVs had no roles in the phosphorylation of Akt in apoptotic NPCs, excluding Pi3k/Akt pathway from the downstream regulators of EVs (Figure S4A,B). Next, we treated EV‐incubated NPCs with 10 μM ERK phosphorylation inhibitor U0126 in the apoptotic model. The inhibitory effects of U0126 on ERK pathway activity were validated by western blotting (Figure 2C,D). TUNEL assay and western blotting results both demonstrated that the anti‐apoptotic effects of A‐EVs were eliminated by U0126, ascertained by the increase of the proportions of TUNEL^+^ cells and the elevation of cleaved Caspase‐3 levels, respectively, in U0126 treatment groups versus their corresponding control groups (Figure 2E‐H). Hence, our observations indicated that the anti‐apoptotic effects of iNPC‐derived EVs are mediated by ERK pathway.

The loss of brain cells, especially NPCs has been considered as a key pathological feature of neurological disorders. NPCs located in the adult subventricular and subgranular zone are attacked by stroke and directed to apoptosis before being induced to migrate to the lesion point and differentiate into neurons for replacing damaged cells.^6^ The excessive expression of apoptotic genes including Bak have been found in various neurodegenerative diseases including AD, inflating apoptosis and causing mortality.^7^, ^8^ Our results demonstrated that iNPC‐derived EVs significantly promoted NPC survival in various apoptosis models, suggesting EVs with a novel role in neuroprotection. However, as all experiments in the study were carried out *in vitro*, the therapeutic effects of iNPC‐EVs on treating neurological disorders need to be verified *in vivo*. Inspiringly, promising results have been obtained by intravenously administrating iNPC‐EVs into middle cerebral artery occlusion (MCAO) mouse models (data are not shown). In addition, ERK signaling, the key downstream regulator of iNPC‐EVs, is one of the most essential intracellular pathways in cell death regulation.^9^ The activation of ERK pathway significantly increases the viability of NPCs in stroke.^10^ However, it is worth‐noting that ERK pathway may exhibit dual roles in controlling the apoptosis of other types of brain cells like neurons.^9^ Thus, the controversial roles of ERK pathway in apoptosis reveal that the anti‐apoptotic effects of iNPC‐derived EVs may not be applicable to all brain cells, which requires more investigations in the future. Besides, multiple research groups including us have reported the important roles of other EV contents, especially miRNAs, in mediating the physiological and pathological effects of EVs in the central nervous system or the peripheral nervous system (see reviews^1^, ^11^, ^12^, ^13^). We have found that WT‐EVs are enriched with miR‐9 and miR‐21.^14^, ^15^ These miRNAs in EVs significantly promote neurogenesis *in vitro*.^14^, ^15^ These findings implied the importance to examine the involvement of iNPC‐EV‐containing miRNAs in the regulation of cell survival, which is under investigation. In summary, our study identified EVs as an essential element of iNPC‐mediated modification of microenvironment and provided new evidence for the inextricable crosstalk between cell extrinsic and intrinsic factors in regulating NPC survival. Our findings not only unveiled a possible mechanism for the therapeutic effect of iNPC transplantation, but also implied iNPC‐derived EVs as a promising cell‐free therapy candidate to protect brain cells in neurological disorders.

## Supporting information

Supporting Information

## Data Availability

The data that support the findings of this study are available from the corresponding author upon reasonable request.
