# Supplementary material for: Induced neural progenitor cell‐derived extracellular vesicles promote neural progenitor cell survival via extracellular signal‐regulated kinase pathway
Source: CNS Neurosci Ther. 2021 Oct 13;27(12):1605–9. doi: 10.1111/cns.13744 (PMC8611769; doi:10.1111/cns.13744)
Supplement: Supplementary file 1 — Supporting Information [file CNS-27-1605-s001.docx]

**Supplemental Information**

**Induced neural progenitor cell-derived extracellular vesicles promote neural progenitor cell survival via extracellular signal–regulated kinase pathway**

**Supplemental Materials**

Supplementary Figure 1

Supplementary Figure 2

Supplementary Figure 3

Supplementary Figure 4

Supplementary Materials and Methods

References

**
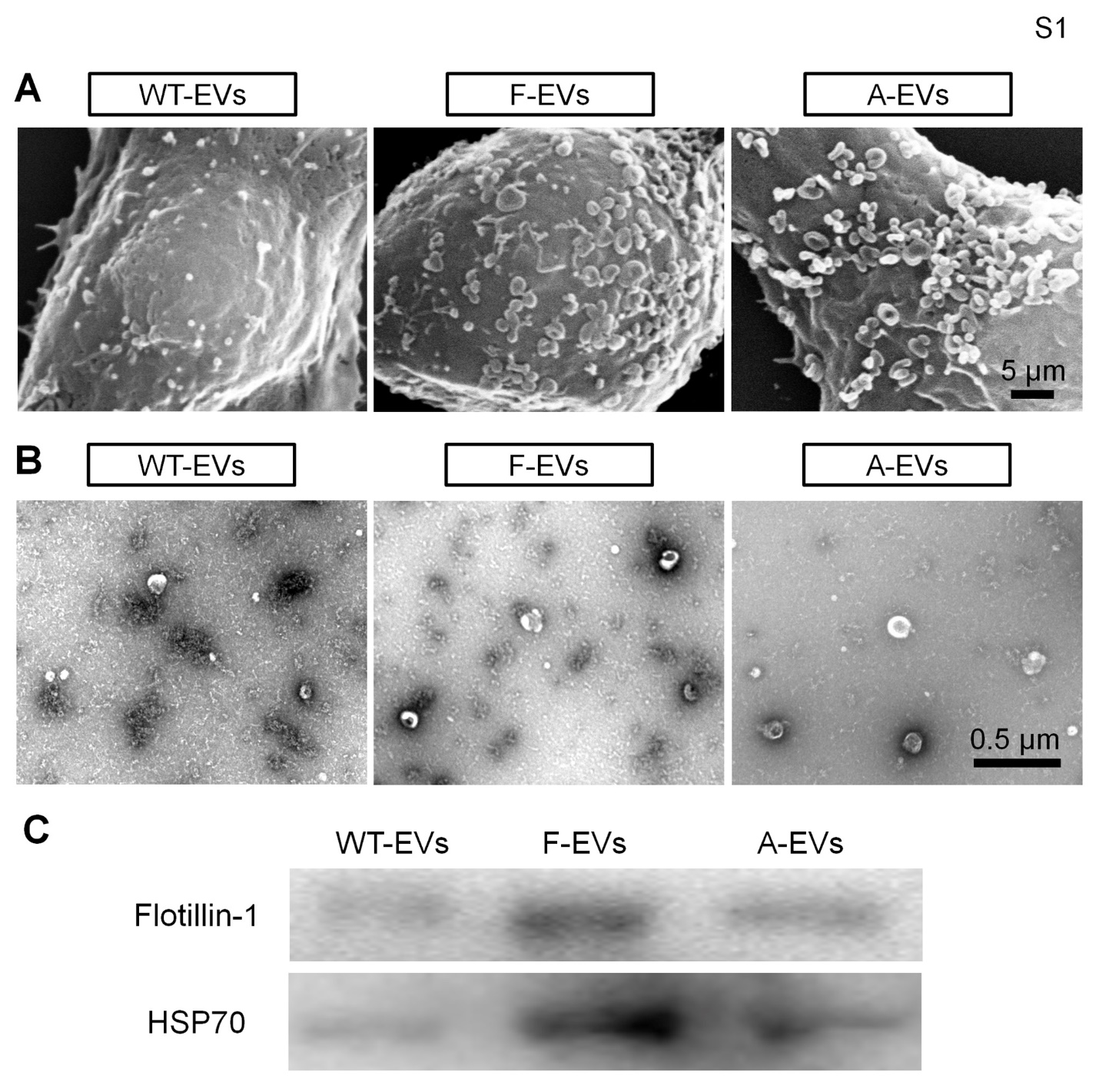
**

**Supplementary Figure 1**. **Characterization of EVs derived from NPCs and iNPCs.**

(**A**) Scanning electron microscopy of WT-EVs, F-EVs, and A-EVs adhering to cell’s surface. (**B**) Transmission electron microscopy characterization of the morphology of WT-EVs, F-EVs, and A-EVs. **C.** Western blot characterization of expressions of EVs markers of WT-EVs, F-EVs, and A-EVs. Scale bar, 5 μm (**A**) and 0.5 μm (**B**).


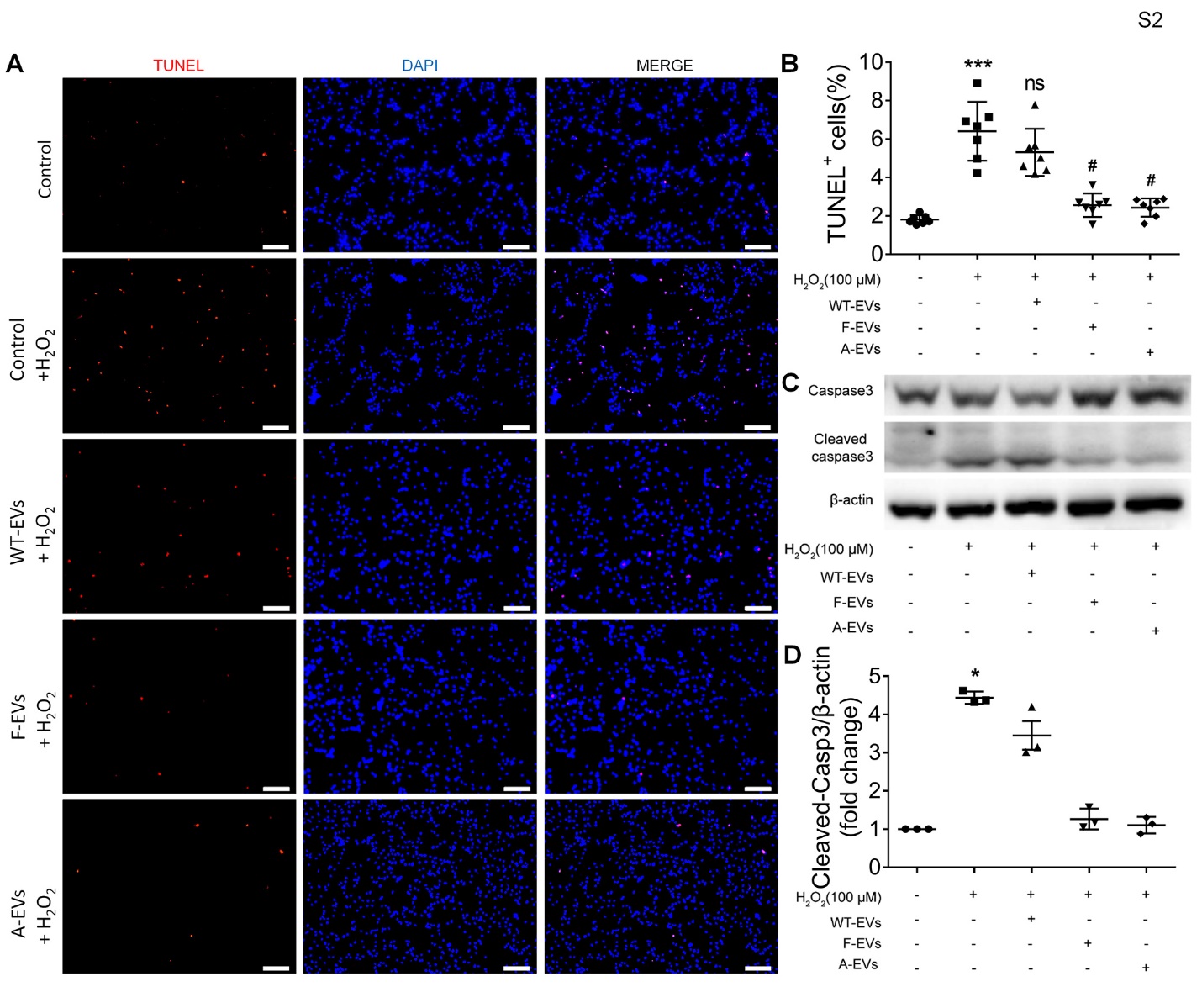


**Supplementary Figure 2. EVs derived from iNPCs inhibit NPC apoptosis under oxidative stress condition.**

(**A**) NPCs were treated with 15 μg/ml EVs for 3 hours in a H_2_O_2_-induced *in vitro* apoptosis model. Representative images of TUNEL (red) and DAPI (blue) staining were shown. (**B**) Quantification of TUNEL^+^ cells (as a percentage of total cells) in the culture. (**C**) The representative western blots showing the expression of cleaved caspase3 and total caspase3 in WT-EV-, F-EV-, and A-EV-treated NPCs under oxidative stress condition. (**D**) Quantification of expression of cleaved caspase3. Results are presented as the mean ± SEM. Scale bar, 100 μm (**A**). * and *** denote *p*<0.05 and *p*<0.001, respectively, in comparison with negative control group. # denotes *p*<0.05 in comparison with H_2_O_2_ treatment group.

**
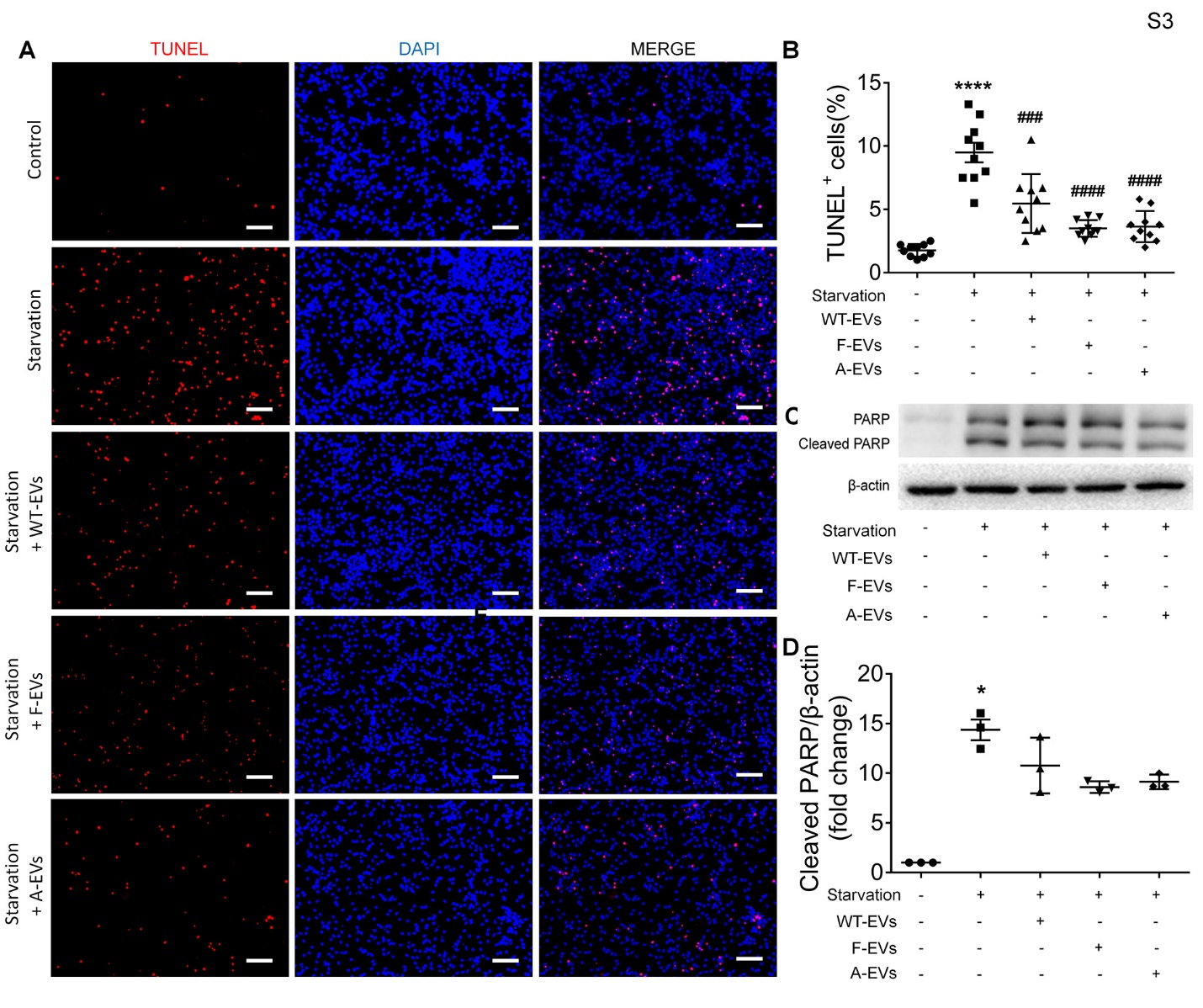
**

**Supplementary Figure 3. EVs derived from iNPCs inhibit NPC apoptosis under nutrient deprivation condition.**

(**A**) NPCs were treated with 15 μg/ml EVs for 12 h in basic culture medium without growth factor supplement. Representative images of TUNEL staining were shown. (**B**) Quantification of TUNEL^+^ cells (as a percentage of total cells) in the culture. (**C**) The representative western blots showing the expression of cleaved PARP and total PARP in WT-EV-, F-EV-, and A-EV-treated NPCs under nutrient deprivation condition. (**D**) Quantification of expression of p-Akt. Results are presented as the mean ± SEM. Scale bar, 100 μm (**A**). * and **** denotes *p*<0.05 and *p*<0.0001 in comparison with negative control group. ### and #### denote *p*<0.001 and *p*<0.0001, respectively, in comparison with starvation group.

**
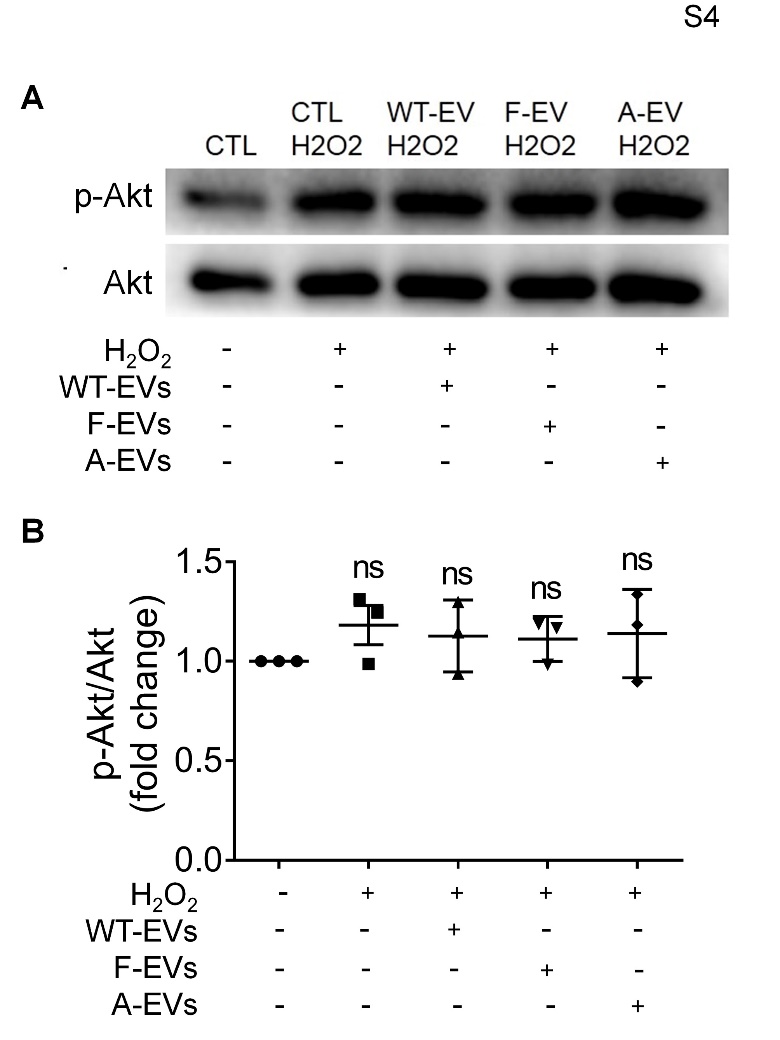
**

**Supplementary Figure 4**. **EVs derived from NPCs and iNPCs have no effects on Akt signaling.**

(**A**) The representative western blots showing the expression of phosphorylated Akt (p-Akt) and total Akt in WT-EV-, F-EV-, and A-EV-treated NPCs under oxidative stress condition. (**B**) Quantification of expression of p-Akt. Results are presented as the mean ± SEM of three independent experiments.

**Supplementary Materials and Methods**

**Materials and Methods**

**The isolation and enrichment of embryonic mouse NPCs**

NPCs were isolated from mouse fetal cortical tissues as previously described [1]. All procedures were conducted according to protocols approved by the Institutional Animal Care and Use Committee (IACUC) of Tongji University School of Medicine (reference number: SYXK (HU) 2014-0026). Briefly, embryonic day 13.5 mouse brain tissues were triturated 15-20 times physically and brain dissociates were filtered through 40 μm filter. Single NPCs were cultured in NPC proliferation medium, containing NeuroCult® NSC Basal Medium (Stem Cell Technologies), NeuroCult® NSC Proliferation Supplements (Stem Cell Technologies), 20 ng/mL FGF2 (BioWalkersville), 20 ng/mL EGF (BioWalkersville), 2 μg/mL heparin (Sigma), N2 supplement, 2 mM L-glutamine, 100 U/ml penicillin & streptomycin for neurosphere formation. Primary neurospheres were then centrifuged at low speed to remove flowing cells in the supernatant, dissociated into single cells with Accutase (Sigma) for 5 minutes at 37 °C, and re-plated for a second round of neurosphere formation. Enriched NPCs were harvested after three rounds of neurosphere formation.

**Collection of EVs**

EVs were isolated from the serum-free culture of NPCs or iNPCs as previously described [2]. Briefly, 6×10^6^ NPCs or iNPCs were cultured in T75 flask with NPC proliferation medium for 24 hours. The conditioned medium was collected and first centrifuged at 300 g for 10 minutes to remove flowing cells, at 3000 g for 20 minutes to remove cellular debris, and then at 10000 g for 30 minutes to remove intracellular organelles. EVs were harvested by ultracentrifugation at 100000 g for 2 hours. All steps of centrifugation were performed at 4 °C.

**Electron microscopy (EM)**

Scanning electron microscopy (SEM): NPCs cultured on glass coverslips were fixed with 2.5% glutaraldehyde and washed three times with PBS. Cells were dehydrated in a series of increasing ethanol concentrations and transferred for critical drying. Cells were then coated with gold-palladium to increase the image contrast and imaged using scanning electron microscope (S-3400, Hitachi).

Transmission electron microscopy (TEM): Purified EVs were negatively stained and then spread on the copper grids. The droplets of EVs were removed with filter paper and air-dried at room temperature (RT). Images were taken using transmission electron microscopy (JEM-1230, JEOL Ltd.).

**Western blotting**

Western blotting was carried out for EV or cell lysates as previously described [2]. Briefly, EVs or cells were lysed in RIPA lysis and extraction buffer (Thermo Scientific). Protein concentration was determined using the BCA (bicinchoninic acid) Protein Assay Kit (Pierce). Blots were incubated with primary antibodies overnight at 4 ℃. The primary antibodies used were as follows: caspase3 (1:1000; Cell Signaling Technologies), cleaved-caspase3 (1:1000; Cell Signaling Technologies), parp (1:1000; Cell Signaling Technologies), β-actin (1:5000; Sigma), flotillin-1 (1:1000; BD biosciences), and HSP70 (1:1000; Cell Signaling Technologies). Corresponding HRP-conjugated anti-goat, anti-rabbit, or anti-mouse (1:10,000, Pierce) secondary antibodies were incubated for 1 hour at RT. Bands were visualized with an ECL kit (Pierce). The density of the immunoblots was determined by image lab software and analyzed using Image J program.

**TUNEL staining**

The cultured cells were planted on coverslips and fixed in 4% formaldehyde for 20 min at RT. Terminal deoxynucleotidyl transferase-mediated dUTP nick end labeling (TUNEL) assay was then performed using TUNEL assay kit (Promega, G3250) according to the manufacturer’s instruction. Images were taken by a Zeiss AX10 fluorescence microscope accompanied with ZEN 2.3 (blue edition) software. For quantification of the percentage of specific cell types in each experiment, cell type-specific antigen positive cells were counted from 15 random fields per group in three coverslips (5 fields each).

**Statistical analyses**

All results are the means of at least three independent experiments ± SEM. Shapiro-Wilk tests were used to evaluate the normality of the distribution. One-way ANOVA or Kruskal-Wallis ANOVA were used for data that are normally distributed or not normally distributed, respectively. Significance was set at *p* < 0.05.

**References**

1. Chen, Q., et al., *CXCR7 Mediates Neural Progenitor Cells Migration to CXCL12 Independent of CXCR4.* Stem Cells, 2015. **33**(8): p. 2574-85.

2. Ma, Y., et al., *Induced neural progenitor cells abundantly secrete extracellular vesicles and promote the proliferation of neural progenitors via extracellular signal-regulated kinase pathways.* Neurobiol Dis, 2018. **124**: p. 322-334.
